# Retraction: *Leishmania donovani* isolates with antimony-resistant but not -sensitive phenotype inhibit sodium antimony gluconate-induced dendritic cell activation

**DOI:** 10.1371/journal.ppat.1013877

**Published:** 2026-01-21

**Authors:** The PLOS Pathogens Editors

Following the publication of this article [1], concerns were raised with results presented in Figs 3, 5–7, and 10. Specifically,

- In Fig 3A, the IκBβ panel appears similar to the IκBε panel when the aspect ratios are adjusted.
- Lanes 1-9 of the OCT-1 panel in Fig 5A appear similar to the OCT-1 panel of Fig 7D.
- Lanes 4-12 of the OCT-1 panel in Fig 6E appear similar to lanes 4-12 in the OCT-1 panel of Fig 7J.
- The AKT panel in Fig 7A appears similar to the IKK2 panel in Fig 7B when flipped.
- In Fig 7J:
  - Lanes 4-14 in the OCT-1 panel appear similar to lanes 16-26 when flipped.
  - There appear to be vertical discontinuities between lanes 18 and 19 in the top panel, and between lanes 15 and 16 in the bottom, OCT-1 panel.
- In Fig 10C:
  - The mγGCS_hc_ IP: rabbit IgG panel appears similar to the mGAPDH: rabbit IgG panel when aspect ratio is adjusted (*i.e.,*: when the top of the mγGCS_hc_ IP: rabbit IgG is cropped off).
  - The mγGCS_hc_ IP: αp50 Ab panel appears similar to the mγGCS_hc_ IP: αp65 Ab panel.

The authors did not comment on the above concerns.

In light of the above concerns, which call into question the reliability and integrity of the published results, the *PLOS Pathogens* Editors retract this article.

All authors agreed with the retraction.

AKH, VY, ES, KKB, and PS apologize for the issues with the published article.
